# Characterization of Bottlenose Dolphin *(Tursiops truncatus)* Sperm Based on Morphometric Traits

**DOI:** 10.3390/biology10050355

**Published:** 2021-04-22

**Authors:** María del Carmen Fuentes-Albero, Silvia Abril Sánchez, José Luis Ros-Santaella, Eliana Pintus, Chiara Luongo, Sara Ruiz Díaz, Carlos Barros García, María Jesús Sánchez Calabuig, Daniel García Párraga, Francisco Alberto García Vázquez

**Affiliations:** 1 Biology Department, Avanqua-Oceanogràfic S.L., 46013 Valencia, Spain; mcarmenfuentesalbero@gmail.com (M.d.C.F.-A.); cbarros@oceanografic.org (C.B.G.); dgarcia@oceanografic.org (D.G.P.); 2 Department of Physiology, Faculty of Veterinary Science, Campus Mare Nostrum, University of Murcia, 30100 Murcia, Spain; silviaabrilsanchez@gmail.com (S.A.S.); chiara.luongo@um.es (C.L.); 3 Department of Veterinary Sciences, Faculty of Agrobiology, Food and Natural Resources, Czech University of Life Sciences Prague, Kamýcká 129, 165 00 Prague, Czech Republic; rossantaella@gmail.com (J.L.R.-S.); eliana.pintus27@gmail.com (E.P.); 4 Department of Animal Reproduction, INIA, 28040 Madrid, Spain; sara.rd.1992@gmail.com (S.R.D.); mariasanchezcalabuig@gmail.com (M.J.S.C.); 5 Department of Medicine and Surgery, Faculty of Veterinary Science, 28040 Madrid, Spain; 6 Research Department, Fundación Oceanogràfic, 46013 Valencia, Spain

**Keywords:** cetacean, morphology, morphometry, semen, sperm cells

## Abstract

**Simple Summary:**

Dolphins are one of the best adapted aquatic mammalians in captivity. While these animals can reproduce naturally in aquaria, many aspects related to their reproduction are still unknown. Their behavior, sociability with humans and ability to learn make dolphins ideal subjects for investigating their reproduction features for conservation programs aimed at this aquatic species and others. It is known that dolphins use a multi-male mating strategy, in which sperm competition could play a fundamental role. This study aims to characterize the shape and dimensions of dolphin sperm from two mature males and putatively classifies them into subpopulations. Moreover, the influence of sex hormone levels (testosterone) and refrigeration (temperature and storage period) with sperm dimension was evaluated. The results indicated that sperm dimensions and shape differed between the two males studied and that the sperm of both males could be classified into two subpopulations depending on their dimensions. Moreover, both testosterone levels and refrigeration were seen to influence sperm dimensions. This investigation provides new insights into sperm competition in dolphin species, and the results could be extrapolated to other endangered aquatic mammalian species.

**Abstract:**

Bottlenose dolphin (*Tursiops truncatus*) males follow many reproductive strategies to ensure their paternity. However, little is known about the sperm traits, including morphometric features, that contribute to their reproductive success. Our aim was to study dolphin sperm morphometry (a total of 13 parameters) in two adult males to evaluate (i) presumptive sperm subpopulations, (ii) the correlation of sperm morphometry with testosterone levels and (iii) the effect of refrigerated storage on the sperm morphometry. Sperm populations were classified into four principal components (PCs) based on morphometry (>94% of cumulative variance). The PCs clustered into two different sperm subpopulations, which differed between males. Furthermore, the levels of serum testosterone were positively correlated with the length of the midpiece but negatively correlated with head width and the principal piece, flagellum and total sperm lengths. Most of the sperm morphometric parameters changed during the storage period (day 1 vs. day 7), but only the principal piece length was affected by the storage temperature (5 °C vs. 15 °C). This is the first study to identify dolphin sperm subpopulations based on morphometry and the influence of serum testosterone and refrigeration on sperm morphometry.

## 1. Introduction

Cetaceans are identified as keystone groups of the marine ecosystem [1], where they play a fundamental role in maintaining holocoenosis stability and integrity [2]. However, human activity, including fishing, maritime traffic, pollution and the degradation of coastal ecosystems [3] are constantly affecting the habitat [4] and health status of cetacean populations [5]. Therefore, their conservation is of great concern, and all cetaceans are protected under Appendix II/Anex A of the Convention on International Trade in Endangered Species of Wild Fauna and Flora (CITES). The bottlenose dolphin (*Tursiops truncatus*) is one of the most closely studied and widely distributed cetaceans. This species is included in the IUCN (International Union for Conservation of Nature and Natural Resources) red list of threatened species [6] (https://www.iucnredlist.org/species/22563/156932432; accessed on 1 March 2021). Keeping bottlenose dolphins in aquariums and the associated research make them an effective model species for understanding cetacean and marine ecosystem health [7]. In this context, aquariums and zoos contribute not only to their ex situ conservation, including recovery programs for sick and/or stranded animals, but also to improving our knowledge of the physiology and anatomy of the species and their reproduction [8].

Considerable variations in reproductive strategies and mating systems have been observed within and between closely related species [9,10]. Male competition for access to females is especially important in delphinids [11]. One of the reproductive particularities of male dolphins is the absence of seminal vesicles and coagulation glands [12], so they cannot produce the vaginal plug that may guarantee paternity after copulation. For this reason, dolphins might have developed other mechanisms to increase the chances of paternity. For example, a large volume of sperm per ejaculation can displace previous ejaculations by other males [13] since the dolphin penis has a conical tip that exerts pressure and expels semen with enough pressure to displace previous fluid in the vagina or in the pseudo-cervix. Male dolphins provide no parental care and appear to mate promiscuously, maximizing the number of receptive females with whom they mate [14]. In this respect, male dolphins are capable of producing consecutive ejaculates while maintaining their quality [15,16], which requires the production of large amounts of sperm in short intervals of time. Consequently, the testicular size of this species has been adapted to be relatively large compared to body size. Indeed, the testes account for more than 0.5% of a male dolphin’s body mass [17,18,19], which is high compared with bulls (0.01%) or humans (0.05%) [17].

This multi-male mating system leads to the need for sperm competition [20], which might be defined as the competition within a single female between the sperm from two or more males to fertilize the oocyte [21]. In mammals, ejaculates may be heterogeneous, presenting several sperm subpopulations [22], which are characterized by differences in motility, DNA fragmentation status, sensitivity to signaling molecules or morphology [23], including different sperm morphometric features [24,25,26]. Heterogeneity refers to differences in the assembly of individual spermatozoa during spermatogenesis, as well as to their differential maturational status at ejaculation in terms of features such as midpiece volume [27] and flagellum length [28,29]. For these reasons, sperm morphometry may determine subsequent effects on swimming speed [30], spermatozoa selection in the uterus [23,31] and the ability to fertilize oocytes [32,33]. Sperm morphometry in different species of dolphin (including bottlenose dolphin) has been described previously (Table 1 [16,34,35,36,37,38]); however, whether sperm morphometry traits influence sperm function has not been studied in this species. The morphometry of sperm subpopulations in other mammals is subject to changes in conditions, including sperm refrigeration or freezing [39]. Such changes can affect sperm motility or morphology, lowering the quality of the semen and its fertilizing capacity [40,41]. Studies in other mammals show that morphometric changes are also related to the concentration of testosterone in the male due to sexual maturity [42] or to seasonality [38,43], since sperm morphometry can adjust to particular social environments [44].

Studies into the sperm morphometry of ejaculate populations may contribute to our knowledge of the reproductive features of certain species of interest for application in conservation programs, including the application to assisted reproductive technologies. To the best of our knowledge, the heterogeneity of dolphin ejaculates in terms of shape and size of the spermatozoa has not been demonstrated previously. Hence, the aim of this work was to determine the sperm morphometric traits of the bottlenose dolphin (*Tursiops truncatus*). To that end, we focus on three objectives: (1) to evaluate the clustering of dolphin sperm ejaculate in subpopulations according to their morphometry; (2) to correlate the serum testosterone levels with sperm morphometry parameters; and (3) to study the effect of sperm refrigeration length and temperature (up to 7 days at 5 °C or 15 °C) on sperm morphometry.

## 2. Material and Methods

### 2.1. Animals

Semen and blood samples were collected from two Atlantic bottlenose male dolphins (*Tursiops truncatus*) (henceforth referred to as Male 1 and Male 2) living in the dolphinarium (*Oceanogràfic-Valencia*, Spain). All the samples were collected from 10:30 a.m. to 12:30 p.m. At the time of the study, Male 1 was ~27 years old and weighed ~195 kg and Male 2 was ~24 years and weighed ~184 kg. Both dolphins have been at the dolphinarium since 2003, so, at the time of the study, they had been living in the same facility for 17 years, and interactions between them were frequent, with Male 1 playing a dominant role over Male 2. Both males have successfully sired several offspring. During the study, the dolphins lived together in an outdoor pool with naturally processed saltwater at ambient temperature (22–27 °C). They were fed a diet of frozen-thawed whole fish (Herring-*Clupea harengus*, Capelin-*Mallotus villosus* and European sprat-*Sprattus sprattus*).

### 2.2. Semen Collection and Processing

The animals were trained for voluntary semen collection. They cooperatively floated with their ventrum above water level and were trained to extrude their penises following a tactile signal. The erect penis was immediately rinsed with saline solution to eliminate potential contamination. Then, very short manual stimulation was performed to induce immediate ejaculation [45], after which the animal was released from this position. The ejaculates (*n* = 11) were collected in sterile plastic bags [45], and, immediately after collection (day 0), divided into two aliquots, avoiding contact with any source of light. One aliquot was not diluted (for experiments 1 and 2), and the second (for experiment 3) was diluted in a 1:1 ratio using a commercial extender (Beltsville Thawing Solution (BTS)) (GVP, Zoitech Lab, Madrid, Spain) warmed at 32 °C. All semen samples (non-diluted and diluted) were sent in a porexpan box to the *Department of Physiology* at the University of Murcia (Murcia, Spain) arriving within 18h of collection at a temperature of 17–20 °C.

### 2.3. Testosterone Experiment

Blood for testosterone testing was collected by puncturing the superficial vessels of the arterio-venous plexus running along each of the lobes of the flukes and transferred to 2 mL lithium heparin tubes (Aquisel^®^, Abrera, Barcelona, Spain) for immediate analysis (maximum elapsed time 1 h). In the laboratory of the Oceanogràfic facilities, the blood samples were centrifuged using standard methods and plasma was carefully pipetted to measure testosterone levels in an ACCES 2 Immunoassay (Beckman Coulter, Brea, CA, USA).

### 2.4. Sperm Morphometric Analysis

**Figure 1:**
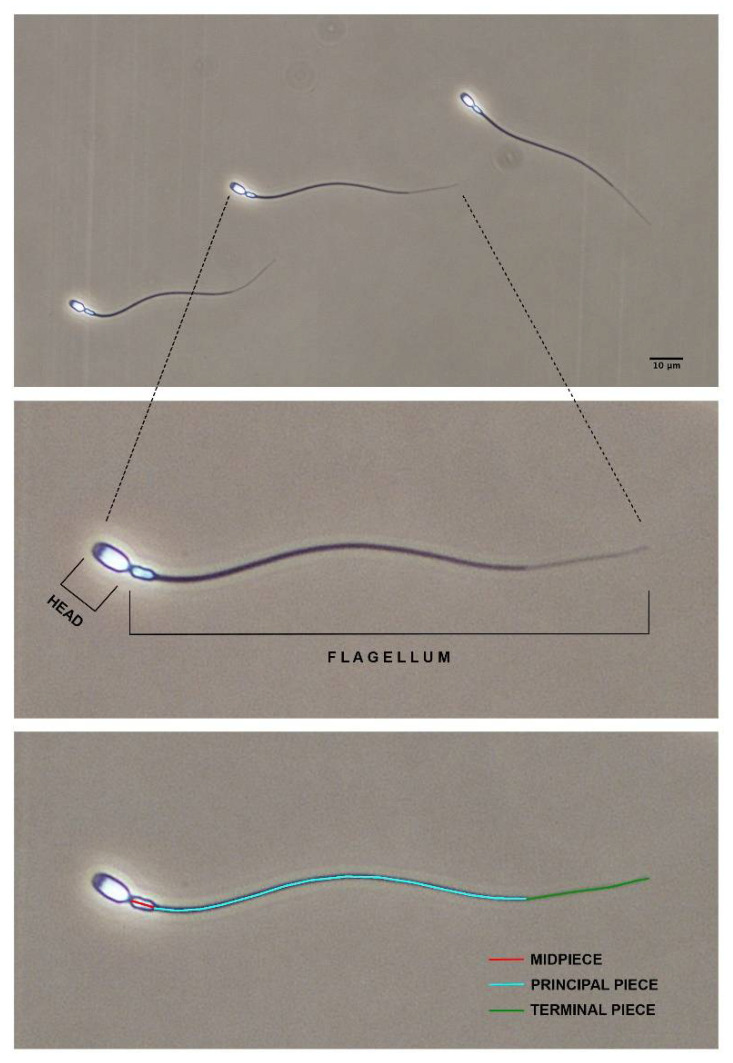
The main structures of bottlenose dolphin spermatozoon (*Tursiops truncatus*): head and flagellum (midpiece, principal piece and terminal piece). The picture was taken under phase-contrast microscopy. Scale bar, 10 µm.

Sperm morphometry was assessed as previously described [46] with minor modifications. Briefly, an aliquot of sperm sample was fixed in 2% glutaraldehyde in phosphate-buffered saline (PBS) solution. A subsample of fixed sperm was used to make the smears, which were air-dried for one day before mounting. Pictures (resolution 2560 × 1920 pixels, TIFF format) were taken using a digital camera (Digital Sight DSFi1, Nikon, Japan) under a phase-contrast microscope (Nikon Eclipse E600, Japan; 40× objective). Pixel size was 0.14 µm in the horizontal and vertical axes. Sperm length was assessed using ImageJ software (US National Institutes of Health, Bethesda, MD, USA). The main structures of a bottlenose dolphin spermatozoon are shown in Figure 1. The following sperm morphometric parameters were determined in 25 spermatozoa per sample: head width (W) (µm), head length (L) (µm), head perimeter (P) (µm), head area (A) (µm^2^), midpiece length (µm), midpiece width (µm), principal piece length (µm), terminal piece length (µm), total flagellum length (µm) and total sperm length (µm). From the head parameters, the following shape factors were also determined, as previously reported [47]: ellipticity (L/W), rugosity (4πA/P^2^) and elongation [(L − W)/(L + W)].

### 2.5. Experimental Design

The morphometry of 875 spermatozoa from a total of 11 ejaculates (6 from Male 1 and 5 from Male 2) was analyzed. The study was conducted by performing three different experiments.

Experiment 1. Analysis of sperm population in bottlenose dolphin ejaculate based on a morphometric study.

The aim of this experiment was to investigate dolphin sperm populations based on morphometric characteristics. For this purpose, a principal component analysis (PCA) was performed, reducing the number of morphometric descriptors to a few standardized variables (principal components-PCs). Then, a statistical analysis (Hopkins Test and Visual Assessment of cluster Tendency-VAT) was carried out to determine whether the sperm population could be clustered. Finally, a clustering method was applied to determine the most suitable number of final clusters. The analysis was performed using a total of 11 ejaculates (6 from Male 1 and 5 from Male 2). A total of 275 spermatozoa were analyzed (25 spermatozoa per ejaculate).

Experiment 2. Correlation of testosterone levels with sperm morphometric variables.

This experiment was performed to assess the relationship between the bottlenose dolphin blood testosterone levels at the moment of semen collection and the different morphometric parameters. The relationship between each PC and testosterone level was also assessed. As in Experiment 1, we used a total of 11 ejaculates (6 from Male 1 and 5 from Male 2), analyzing a total of 275 spermatozoa (25 spermatozoa per ejaculate).

Experiment 3. Effect of storage period on dolphin sperm morphometry during refrigeration.

The aim of this experiment was to evaluate differences in the morphometry of dolphin spermatozoa between semen samples during refrigeration (days 1 and 7 of storage) at two different temperatures (5 °C and 15 °C). Male 1 and Male 2 were used for this analysis (*n* = 6). The morphometry of a total of 600 spermatozoa was evaluated (300 cells per male), and 25 spermatozoa per temperature and day of storage were evaluated.

### 2.6. Statistical Analysis

In experiment 1, a total of 275 spermatozoa were included for the analysis of sperm subpopulations, using the data obtained from each dolphin by means of clustering. To minimize the number of parameters for clustering procedures, a PCA of the morphometric data was performed using the free statistical software R. PCA allows a large number of variables to be summarized in fewer jointly uncorrelated principal components (PCs), with the result being considered good when there are few PCs accounting for a large proportion of the total variance. Only the PCs with an eigenvalue (variance extracted for that particular PC) greater than 1 (Kaiser criterion) were selected. Two methods were followed to evaluate the clustering tendency (free statistical software R): a statistical method using the Hopkins test (H value) and a visual method using a Visual Assessment of Tendency (VAT) algorithm [48]. Clustering was considered significant when H values were under 0.5. The clValid package [49] was used to select the optimal clustering algorithms and the free software R to select the optimal number of clusters. The analysis was performed by simultaneously computing internal cluster measurements (Connectivity, Silhouette width, and Dunn index) for multiple clustering algorithms (hierarchical, K-means, and Partitioning Around Medoids (PAM)) in combination with a range of cluster numbers. The optimal clustering algorithm picks the cluster with the lowest value for Connectivity and the highest values for Silhouette width and Dunn index (Supplementary Figure S1). The optimal cluster number was chosen based on the same criteria used in optimal clustering algorithms (Supplementary Figure S1). Finally, clustering was performed using hierarchical algorithms by complete linkage and the Euclidean clustering metric using R [48]. Agglomerative hierarchical clustering was plotted by heatmap using the “dendextend” R package. All data were scaled before the clustering analysis.

For experiment 2, the Shapiro–Wilk test was used to evaluate the normal distribution of the residuals of all morphometric variables and testosterone. Since several sperm morphometric variables (principal piece length, terminal piece length, flagellum length, total sperm length, PC1 and PC2) and testosterone were not normally distributed, the correlation between the morphometry variables from 11 ejaculates (*n* = 275 spermatozoa analyzed/25 sperm per ejaculate) and testosterone plasma hormone was assessed by Spearman’s correlation coefficient. The statistical analysis for experiment 2 was performed using SAS University Edition (SAS, 2016).

In experiment 3, the effect of storage period (1 and 7 days) and temperature (5 and 15 °C) on sperm morphometry (*n* = 600) was evaluated. Sphericity for repeated measurements was assessed using the restricted likelihood ratio test Huynh–Feldt and Greenhouse–Geisser covariance structures. If the difference between these tests (distributed under the null hypothesis as a χ^2^ with the difference between the degrees of freedom, df) was greater than χ^2^ df, the sphericity of the data was considered (all the morphometric variables of the spermatozoa confirmed the sphericity of the data). The above variables were analyzed using Proc Mixed procedures. The model included the storage period (1 and 7 days), the temperature (5 or 15 °C), and the interaction between these as the main effect, with the different samples and males as the random effect. A first-order autoregressive covariance structure was used to adjust the difference in data according to the differences with time, and the Tukey post-hoc analysis was applied to detect differences between experimental groups. Differences were considered statistically significant when *p* < 0.05. The statistics for experiment 3 were performed by SAS University Edition (SAS, 2016).

## 3. Results

Experiment 1. Analysis of sperm population in bottlenose dolphin ejaculate based on morphometric study.

### 3.1. Development of Sperm Morphometric Variables as Principal Components (PCs)

The mean values for the dimensions of the spermatozoa from both males of the study are shown in Table 2. PCA revealed that four PCs accounted for more than 91% of the cumulative variance, PC1 providing the highest explained variance (47.21%), followed to PC2 (25.65%), PC3 (11.17%) and PC4 (7.72%) (Table 3). The PCs were obtained from weighing variables against their eigenvectors, and the morphometric variables were grouped into the principal components (PC1, PC2, PC3 and PC4) according to the highest value of their eigenvector (variables in parentheses; see Table 3). PC1 was related with parameters of sperm length (head, principal piece, terminal piece and total sperm length); PC2 was related with parameters of sperm head shape (width, ellipticity, rugosity and elongation); PC3 with parameters of head, midpiece and flagellum size (head area, head perimeter, midpiece length and flagellum length); and PC4 was related with midpiece width (Table 3).

### 3.2. Assessment of Data Clustering Tendency

**Figure 2:**
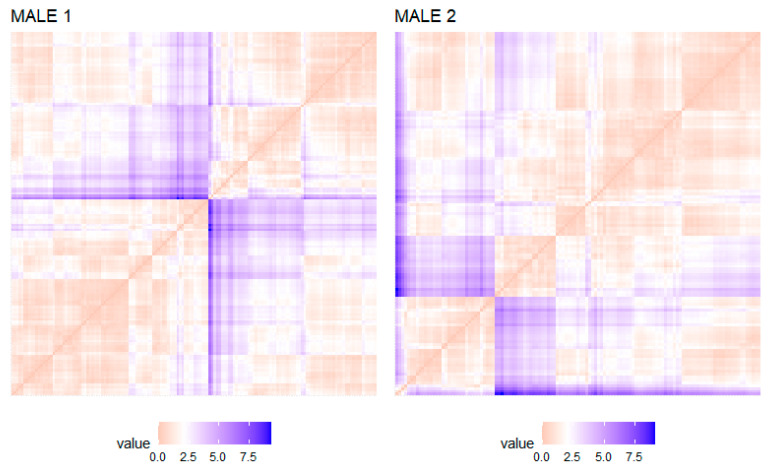
Quality of clustering based on Visual Assessment of the cluster Tendency (VAT) in sperm population of two bottlenose dolphin (*Tursiops truncatus*) (Male 1—left panel; Male 2—right panel). The cuvette visually detects the clustering trend by counting the number of square orange blocks along the diagonal in a cuvette image. Orange indicates high similarity (values close to 0), while blue indicates low similarity (values higher than 2.5).

Evaluation of the clustering trend consisted of examining whether the data could be divided into clusters together. The analysis indicated that the morphometric data of both males were clusterable (H value of male 1 = 0.22 and male 2 = 0.21, which are far below the threshold 0.5). Based on the VAT method, the clustering tendency was also visually assessed by graphically counting the number of orange squares along the diagonal of a dissimilarity matrix in the VAT graph (Figure 2). The orange squares illustrate data with a high similarity (values close to 0), while the blue squares illustrate data of low similarity (values higher than 2.5). The image (Figure 2) identifies areas of similar and dissimilar squares along the diagonal in both males, as indicated by the orange areas in the cuvette. Therefore, both the Hopkins test and VAT indicated that the data are clusterable in accordance with the morphometric PCs.

### 3.3. Clustering Algorithms and Cluster Numbers

The cluster analysis was carried out by comparing three clustering algorithms (hierarchical, K-means and PAM) based on Connectivity, Silhouette width and Dunn index measures (Supplementary Figure S1a). The hierarchical clustering algorithm was the best in each case (for Connectivity, Silhouette width and Dunn index measures) for both males, as the score for Connectivity was lower in the hierarchical algorithms than in K-means and PAM algorithms, and the score for Silhouette width and Dunn index measures were higher in the hierarchical algorithms than in the other algorithms in both males. The optimal number of clusters was two based on the three measures, regardless of the clustering algorithm in both males (Supplementary Figure S1).

### 3.4. Hierarchical Clustering in a Heatmap

**Figure 3:**
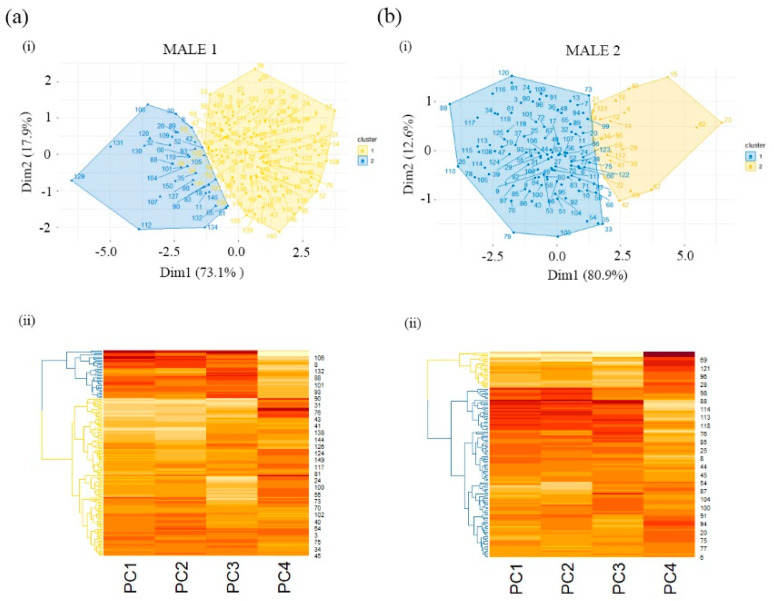
Bottlenose dolphin (*Tursiops truncatus*) sperm clustering. (**i**) Graphs showing two cluster areas (blue for cluster 1 and yellow for cluster 2) of sperm populations in both males of the study (**a**,**b**). The numbers indicate individual sperm classified in the clusters. (**ii**) Hierarchical tree/dendrogram combined with heatmap. The dendrograms indicate the relationships between the principal components (columns) and spermatozoa (rows) based on Spearman’s correlation coefficients. Each leaf corresponds to one spermatozoon. As we move up the tree, spermatozoa that are similar to each other are combined into branches, which are themselves fused at a higher height. Horizontal axis shows the height of the fusion that indicates the dissimilarity or similarity distance between two spermatozoa. Similarity decreases with the height of fusion. Heatmaps show the columns/rows of the data matrix reordered according to the hierarchical clustering result, placing similar spermatozoa close to each other based on principal components (PC1, PC2, PC3 and PC4). Spermatozoa similarity or dissimilarity is indicated by an orange scale. Dark orange indicates high similarity; light orange indicates low similarity.

A clustering hierarchical tree (dendrogram) and heatmap from morphometric PC variables are depicted in Figure 3. Two clusters (blue and yellow areas in the graph) were identified in both males (Figure 3a(i),b(i)). The heatmaps provided similar results for spermatozoa similarity and dissimilarity to each cluster in Males 1 and 2 (Figure 3a(ii),b(ii)). In both males, cluster 1 (blue dendrogram) placed spermatozoa similarity in PC1, PC2 and PC3 and spermatozoa dissimilarity in PC4, and cluster 2 (yellow dendrogram) had spermatozoa dissimilarity in PC1, PC2 and PC3 and spermatozoa similarity in PC4 (Figure 3a(ii),b(ii)).

Experiment 2. Correlation of testosterone levels with sperm morphometric variables.

The correlations of different sperm morphometry variables and testosterone are shown in Table 4. Midpiece length and PC2 showed a positive correlation with testosterone levels (r = 0.68 and *p* = 0.02; r = 0.63 and *p* = 0.04, respectively), while head width, principal piece length, flagellum length and total sperm length were negatively correlated with testosterone concentrations in blood plasma (r ≥ −0.60; *p*< 0.05). None of the other variables were correlated with testosterone concentrations, including PC1, PC3 and PC4.

The results of spermatozoa morphometry during refrigeration (5 °C and 15 °C) from day 1 to day 7 are summarized in Table 5. The head width, head area, head perimeter, head rugosity, midpiece length, flagellum length and total sperm length decreased from day 1 to day 7 (*p* < 0.05), while head ellipticity and elongation increased over the same period (*p* = 0.04). However, head length, midpiece width, principal piece length and terminal piece length did not change with time. None of the parameters (except principal piece length) differed with the different refrigeration temperatures (*p* > 0.05), and there was no interaction between time and temperature. However, for a given time, the principal piece length was higher at 5 °C than 15 °C (*p* = 0.02).

## 4. Discussion

Dolphins represent one of the cetaceans that are under protection. The fact that many of these animals are in aquariums opens up the possibility of studying their reproductive features for application in future assisted reproductive techniques that can be used for the conservation of this species and cetaceans in general. In this respect, the study of sperm populations and behavior in different conditions is of interest to increase knowledge of sperm traits. Although sperm morphometry has been studied in several mammals, including carnivores [50,51], ruminants [52,53], camelids [54] and humans [26], the effect of changes in sperm size and shape on sperm functions is still poorly understood. The results of this study show, for the first time, the complete sperm morphometry and sperm subpopulations based on morphometry for any species of dolphin. Moreover, the study demonstrates how some morphometric variables (e.g., midpiece and flagellum length) are influenced by the level of blood testosterone or storage period but are barely affected by the temperature of refrigerated storage. However, we have to consider the low number of males included in the study (only two) due to the difficulties in obtaining fresh semen samples from a higher number of males.

The morphometric parameters observed in the present study are similar to previous findings for bottlenose dolphin sperm (see Table 1 [16,34,35,36,37,38]) and comparable to those for other cetaceans [35]. Knowledge of sperm dimensions may provide important information on the evolutionary adaption of spermatozoa [55,56], and the standardization of sperm features in captivity animals may provide information of interest when comparing the same features in wild animals, because external factors (e.g., environmental pollutants) might modify sperm dimensions [57]. As mentioned earlier, the bottlenose dolphin has a multi-male mating system. Thus, sperm competition implies high-quality sperm production, with a large volume of ejaculate, high motility and a minimal percentage of morphoanomalies [58]. The morphological characteristics of bottlenose dolphin spermatozoa, first described by Fleming et al. [34], are in line with the seminal parameters widely described for this species [59,60,61]. The long length of the flagellum and the extremely streamlined design of the spermatozoa may contribute to the high motility rates observed previously in this species [16]. The midpiece length is very short compared with other mammals, but it is large in volume (estimated at 3.9 µm^3^, calculated using the volume of a cylinder). This volume is correlated with the presence of 6–8 very large mitochondria with extensive mitochondrial cristae [16]. In fact, larger midpiece volume is known in species with multiple partner systems, which result in higher sperm motility and thus in successful sperm competition [62]. In addition, the bottlenose dolphin sperm head is small compared with that of other mammals, suggesting differences in the organization and/or compaction of nuclear chromatin. Moreover, the head presents edges parallel to the longitudinal axis in the post-acrosomal region, which may be related to the fertilization process [34,35].

Morphometrically differing sperm head subpopulations have been identified in several mammalian species [26,38,51,52,63,64]. Such subpopulations have been related with sensitivity to cryopreservation, leading in cases to a reduction in sperm head size [41,50] or even correlated with fragmented DNA [64]. Nevertheless, dolphin sperm subpopulations have only been described in connection with lectin patterns [65] and kinetic variables [16]. In the present study, the types of sperm produced by both males had different traits, as observed by the clusterization of the sperm population, suggesting that a given male may produce a mix of different sperm with different functions. It has previously been proposed that the different sperm phenotypes of a seminal sample may have different fertile potentials, providing advantages at different stages of the fertilization process [66]. Although the importance of sperm morphology (including morphometry) during female tract trajectory has been studied in other species [23,31,67], despite its undeniable interest, there is a lack of information regarding the role of sperm morphology on competition in dolphins. Previous studies in rodents have been performed in monogamous vs. polygamous species, finding that sperm competition seems to affect different reproductive traits, including sperm morphometries [68]. The present study opens up a new approach for studying the role of sperm morphometry in sperm function in multi-male mating species, allowing, among other things, better identification of the males with different sperm cryoconservation ability offering better cyroconservation possibilities, as previously suggested [69]. In this respect, our results showed that the sperm populations of both males were clusterizable into two subpopulations. These subpopulations, however, differed between males. As mentioned above, Male 1 was higher in the social hierarchy than Male 2, which may have induced the production of different sperm traits suggesting a competitive advantage of the sperm of Male 1. Higher sperm midpiece volumes have been observed in primate species in which the females mate with multiple partners than in those species in which they mate with only one partner [62]. The sperm midpiece contains mitochondria, whose volumes have been positively correlated with ATP content and sperm swimming velocities [70]. However, a cautionary note must be added to the assumptions of our study due to the low number of males included.

In bottlenose dolphin, differences in testosterone levels depending on the season have been reported [71]. The high quality of ejaculated dolphin sperm [16,72], together with the existence of sperm subpopulations (based on morphometry and variations according to the testosterone levels) may be adaptations for sperm competition and linked to the mating system of this species. However, the potential links between testosterone and sperm traits, including morphometry, are not clear, although a relationship between testosterone levels and normal spermatozoa has been observed in other species [73]. In our study of bottlenose dolphin, a positive correlation was observed between smaller sperm head size and higher testosterone concentration. Differences in spermatozoa head morphometry could be related to the influence of environmental factors and genetic effects [74]. Additionally, sperm head size may be related to fertility, as previously described in boar, where high-fertility individuals were seen to have significantly smaller and less elongated sperm heads than low-fertility males [75]. However, a study conducted in rabbit concluded that sperm head morphometry is not related to fertility [76]. Interestingly, the same authors showed that spermatozoa head size may be heritable [74]. A study conducted in epididymal cat sperm reported that sperm DNA fragmentation is not correlated with sperm head morphometry [77] or with chromatin integrity, as also reported in boar spermatozoa [47]. Additionally, there are significant differences in sperm head morphometry between boar breeds, individuals and even among ejaculates [47], as also occurs in stallions [78], and in different portions of the same ejaculate [63]. Moreover, in ram, ejaculates showing differences in sperm motility and membrane integrity have been related to specific morphometric subpopulations, including an important seasonal effect on sperm morphometric traits [79]. Although we found a correlation between sperm head size and testosterone levels in bottlenose dolphin, there is still a lack of information in the literature regarding the relationship between these parameters. Additionally, our data demonstrated a shorter flagellum and longer midpiece when testosterone levels were high. Taking these two parameters together (midpiece and flagellum length), it has been reported that a longer midpiece and shorter flagellum may be adaptive in a stressful or competitive social environment [44], including taking part in breeding season or even influencing sperm longevity [80]. Accordingly, the size of the sperm midpiece is expected to increase in response to sperm competition to provide the cell with more energy [27] since the size of the midpiece is an indicator of mitochondrial loading, which provides energy to the spermatozoa [62,81] and is positively correlated with competitive ability and swimming performance [82]. Furthermore, other authors [44] have reported that individual changes in bird sperm midpiece and flagellum length were significantly related to individual changes in testosterone levels.

Sperm morphometric characteristics might help explain sperm functionality during storage [69], which is important for semen conservation in bottlenose dolphin. Our results showed that cool storage reduces sperm head dimensions (width, area, perimeter) and flagellum (midpiece and flagellum length). In boars, it has been shown that some morphometric characteristics of the sperm head can be affected by the cooling process [83,84], suggesting that changes in sperm morphometry during refrigeration-storage might be caused by the oxidative stress that provokes deterioration of the semen sample [83]. This is consistent with previous studies in several species, in which a long-term conservation process, such as cryopreservation, decreased the dimension of the spermatozoa head [50,85,86]. Likewise, it is known that the sperm cooling process, including cryopreservation, affects acrosome membranes [87,88], which could explain the reductions observed in head dimensions; however, the acrosome integrity in chilled dolphin sperm is well conserved during the storage period [72]. Therefore, another plausible hypothesis could be the association of sperm morphometry with DNA integrity. Nevertheless, there is certain controversy related to this issue. Some studies have demonstrated a relationship between changes in the sperm head and DNA integrity [89,90,91], although others found no such association. Our results also show that storage produced a decrease in other sperm morphometry parameters, including midpiece length. As mentioned above, the midpiece is characterized by the presence of mitochondria, which provide energy to spermatozoa, improving their swimming velocity [16,70] and increasing longevity [92]. A previous study described a decrease in dolphin sperm motility with time [72]. Thus, since midpiece length is associated with sperm motility, a decrease in this morphometric parameter is to be expected. Regarding the principal piece length, this parameter is also involved in the maintenance of sperm motility [93]. Our results showed that the principal piece length was greater at 5 °C than at 15 °C. This suggests that sperm velocity would be better at 5 °C, as suggested by previous studies that found that higher sperm motility resulted from lower temperatures [60,72]. Therefore, refrigeration could be an interesting alternative to cryopreservation in order to avoid morphometric alterations and to maintain high levels of motility and viability, and to ensure the good morphology of bottlenose dolphin sperm [72].

## 5. Conclusions

This is the first description of sperm populations in *Tursiops truncatus* samples based on morphometry. Bottlenose dolphin sperm were grouped by morphometric features, which differed between males. Moreover, a relationship (positive and negative) between blood testosterone level and some sperm morphometric characteristics was asserted. Finally, a cool-storage period, but not the temperature of refrigeration, influenced head and flagellum size and head shape. It has to be considered as well that there was a low number of males used in this study (only two) due to the difficulty of obtaining fresh semen samples from a higher number of males. Therefore, further studies should be addressed to increase the number of male dolphins and go deep in the reproduction features analysis of this species.

## Figures and Tables

**Table 1:** Comparative table of sperm morphometric parameters reported in different dolphin species.

		Species (Reference)					
Sperm	Parameters	Atlantic Bottlenose Dolphin (Fleming et al., 1981) [34]	Pacific White-Sided Dolphin (Miller et al., 2002) [35]	Humpback Dolphin, Long-Beaked Common Dolphin, Rough Tooth Dolphin (Downing Meisner et al., 2005) [36]	Amazon River Dolphin (Amaral et al., 2017) [37]	Atlantic Bottlenose Dolphin (van der Horst et al., 2018) [16]	Atlantic Bottlenose Dolphin (O’Brien et al., 2019) [38]
Head	Width (µm)	2.0	1.4 ^a^; 1.9 ^b,c^	1.9–2.0	2.2	2.2–2.5	2.7
	Length (µm)	4.5	3.5 ^a,b^; 3.8 ^c^	3.9–3.6	5.6	4.7–5.0	4.4
	Area (µm^2^)	--	--	--	--	9.1–10.7	7.1
	Perimeter (µm)	--	--	--	--	12.1–13.1	9.2
	Ellipticity	--	--	--	--	1.9–2.2	--
	Rugosity	--	--	--	--	0.7–0.8	--
	Elongation	--	--	--	--	--	--
Flagellum	Midpiece width (µm)	--	0.6 ^a,b,c^	--	1.2	1.1–1.2	--
	Midpiece length (µm)	4.0	2.3 ^a,c^; 2.5 ^b^	--	3.3	3.5	--
	Principal piece length (µm)	--	--	--	--	--	--
	Terminal piece length (µm)	--	--	--	--	--	--
	Flagellum length (µm)	60.0	60.5 ^a^; 59.8 ^d^	--	52.9	60.0	--
Total sperm length (µm)		65.0	62–68 ^d^	--	62.3	65.0	--
Observations		Frozen-thawed ejaculated sperm	Epididymal sperm	Fresh ejaculated sperm	Fresh ejaculated sperm	Fresh ejaculated sperm The values represent a range between three dolphins	Epididymal sperm
Evaluation technique		Scanning Electron Microscopy	^a^ Negatively stained sperm ^b^ Scanning Electron Microscopy ^c^ Ultrathin-sectioned sperm ^d^ Light Microscopy	Scanning Electron Microscopy	Phase-contrast Microscopy	Automated Sperm Class Analyzer using SpermBlue stain	Automated Sperm Class Analyzer using Hemacolor stain

**Table 2:** Sperm morphometric parameters in bottlenose dolphin (*Tursiops truncatus*). SD = standard deviation. *p* < 0.05 indicates statistical differences between males.

		Male 1		Male 2		Average		
Sperm	Parameters	Mean	SD	Mean	SD	Mean	SD	*p*-Value
Head	Width (µm)	2.48	0.12	2.53	0.12	2.51	0.12	ns
	Length (µm)	4.96	0.22	5.29	0.19	5.12	0.21	<0.0001
	Area (µm^2^)	9.67	0.67	10.53	0.57	10.10	0.62	0.004
	Perimeter (µm)	12.01	0.43	12.68	0.36	12.35	0.39	<0.0001
	Ellipticity	2.00	0.12	2.09	0.13	2.05	0.13	<0.0001
	Rugosity	0.84	0.03	0.82	0.03	0.83	0.03	<0.0001
	Elongation	0.33	0.03	0.35	0.03	0.34	0.03	<0.0001
Flagellum	Midpiece width (µm)	1.30	0.11	1.29	0.11	1.30	0.11	ns
	Midpiece length (µm)	3.11	0.25	2.86	0.22	2.99	0.23	<0.0001
	Principal piece length (µm)	45.98	1.28	53.66	1.13	49.82	1.20	<0.0001
	Terminal piece length (µm)	15.05	1.14	10.75	0.72	12.90	0.93	<0.0001
	Flagellum length (µm)	64.15	1.11	67.27	1.22	65.71	1.17	<0.0001
Total sperm length (µm)		69.10	1.16	72.56	1.22	70.83	1.19	<0.0001

Ns = no significance.

**Table 3:** Analysis of principal components (PCA) performed with bottlenose dolphin (*Tursiops truncatus*) sperm morphometry data. Eigenvalues and variance explained in PC1, PC2, PC3 and PC4 and eigenvectors from the sperm morphometric variables distributed in four PCs. PC1 refers to sperm length; PC2 to shape of sperm head; PC3 to size of head, midpiece and flagellum; and PC4 refers to midpiece width.

	Parameters	PC1	PC2	PC3	PC4
Eigenvalues		2.48	1.83	1.21	1.00
Variance Explained (%) *		47.21	25.65	11.17	7.72
Cumulative Proportion (%)		47.21	72.86	84.03	91.74
Eigenvectors ^†^	Head width	0.04	(0.51)	0.29	0.01
	Head length	(0.37)	−0.06	0.33	−0.03
	Head area	0.27	0.29	(0.42)	−0.01
	Head perimeter	0.35	0.10	(0.39)	−0.02
	Head ellipticity	0.26	(−0.42)	0.05	−0.03
	Head rugosity	−0.26	(0.42)	−0.05	0.03
	Head elongation	0.26	(−0.42)	0.05	−0.03
	Midpiece width	−0.01	−0.04	0.06	(0.99)
	Midpiece length	−0.16	−0.22	(0.40)	0.07
	Principal piece length	(0.35)	0.16	−0.29	0.03
	Terminal piece length	(−0.31)	−0.14	0.17	0.03
	Flagellum length	0.32	0.12	(−0.34)	0.10
	Total sperm length	(0.35)	0.11	−0.28	0.09

Eigenvectors in parentheses were chosen for each PC. * Variance explained is the proportion of the total variance explained by each PC. ^†^ The eigenvectors are a measure of association of the original parameters with the resulting PCs.

**Table 4:** Correlation between bottlenose dolphin (*Tursiops truncatus*) sperm morphometry variables and blood testosterone concentrations. A statistical correlation was considered when *p* < 0.05.

Sperm	Parameters	Spearman’s Correlation	*p*-Value
Head	Width	−0.62	0.04
	Length	−0.37	0.27
	Area	−0.57	0.07
	Perimeter	−0.44	0.17
	Ellipticity	−0.14	0.68
	Rugosity	0.04	0.90
	Elongation	−0.09	0.79
Flagellum	Midpiece width	0.00	1.00
	Midpiece length	0.68	0.02
	Principal piece length	−0.65	0.03
	Terminal piece length	0.37	0.26
	Flagellum length	−0.60	0.04
Total sperm length		−0.62	0.04
PCs	PC1	0.53	0.09
	PC2	0.63	0.04
	PC3	0.53	0.09
	PC4	−0.49	0.12

Experiment 3. Effect of storage period on dolphin sperm morphometry during refrigeration.

**Table 5:** Bottlenose dolphin (*Tursiops truncatus*) morphometric sperm parameters comparison after 7 day refrigeration period (day 1 vs. day 7). Data are expressed as the mean and SEM. Values within a row with *p* < 0.05 were considered statistically different.

		Time			
Sperm	Parameters	Day 1	Day 7	SEM	*p*-Value
Head	Width (µm)	2.45	2.42	0.06	0.002
	Length (µm)	5.04	5.06	0.18	ns
	Area (µm^2^)	9.74	9.59	0.58	0.001
	Perimeter (µm)	12.16	12.09	0.40	0.04
	Ellipticity	2.07	2.09	0.03	0.04
	Rugosity	0.83	0.82	0.01	0.04
	Elongation	0.34	0.35	0.01	0.04
Flagellum	Midpiece width (µm)	1.26	1.25	0.02	ns
	Midpiece length (µm)	2.95	2.87	0.07	<0.001
	Principal piece length (µm)	49.73	49.58	3.93	ns
	Terminal piece length (µm)	12.60	12.49	2.25	ns
	Flagellum length (µm)	65.28	64.94	1.61	0.001
Total sperm length (µm)		70.35	69.99	1.80	0.001

ns = no significance.

## Data Availability

All data generated or analyzed during this study are included in the published paper.

## Supplementary Materials

The following are available online at https://www.mdpi.com/article/10.3390/biology10050355/s1, Figure S1. (a) Validation clustering algorithms (Hierarchical, K-means and PAM) and cluster numbers by internal measures (Connectivity, Silhouette width and Dunn index) in the two males of the study and (b) graphic showing the optimal number of clusters as average silhouette width in both males.
