# Supplementary material for: Characterization of Bottlenose Dolphin (Tursiops truncatus) Sperm Based on Morphometric Traits
Source: Biology (Basel). 2021 Apr 22;10(5):355. doi: 10.3390/biology10050355 (PMC8143526; doi:10.3390/biology10050355)
Supplement: Supplementary file 1 [file biology-10-00355-s001.zip › biology-1168569-supplementary.pdf]

(a)

Validation Measures	Male 1					Male 2				
	Cluster number					Cluster number				
	2	3	4	5	6	2	3	4	5	6
**Hierarchical**										
Connectivity	**11.03**	13.81	16.74	32.10	33.14	**11.46**	19.21	23.28	26.34	26.65
Dunn	**0.17**	0.17	0.17	0.11	0.11	0.12	**0.15**	**0.15**	**0.15**	**0.15**
Silhouete	**0.48**	0.43	0.20	0.29	0.29	**0.48**	0.37	0.31	0.23	0.22
**K-means**										
Connectivity	**17.02**	38.41	48.50	73.19	74.79	**19.84**	47.61	37.79	55.60	73.01
Dunn	0.08	0.07	0.06	0.08	**0.10**	0.06	0.06	**0.11**	**0.11**	0.04
Silhouete	**0.37**	0.30	0.27	0.26	0.27	**0.38**	0.33	0.34	0.28	0.25
**PAM**										
Connectivity	**18.88**	43.51	57.39	71.35	76.69	**22.73**	43.60	54.07	70.30	75.05
Dunn	0.05	**0.07**	0.04	0.04	0.05	0.04	0.06	0.06	**0.11**	0.06
Silhouete	**0.37**	0.28	0.26	0.26	0.27	**0.38**	0.31	0.25	0.25	0.25

PAM: Partitioning Around Medoids.

(b)

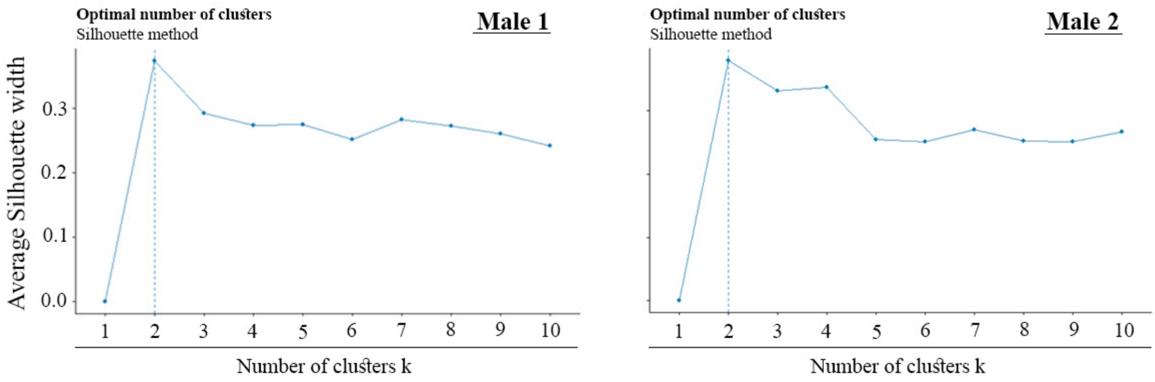

Figure S1. (**a**) Validation clustering algorithms (Hierarchical, K-means and PAM) and cluster numbers by internal measures (Connectivity, Silhouette width and Dunn index) in the two males of the study and (**b**) graphic showing the optimal number of clusters as average silhouette width in both males.
